# Varicella-Zoster-Mediated Radiculitis Reactivation following Cervical Spine Surgery: Case Report and Review of the Literature

**DOI:** 10.1155/2013/647486

**Published:** 2013-10-22

**Authors:** Doniel Drazin, George Hanna, Faris Shweikeh, Sunil Jeswani, Leah Lovely, Richard Sokolov, John C. Liu

**Affiliations:** ^1^Department of Neurosurgery, Cedars-Sinai Medical Center, Los Angeles, CA 90048, USA; ^2^Department of Medicine, Cedars-Sinai Medical Center, Los Angeles, CA 90048, USA

## Abstract

Varicella-zoster virus and herpes simplex virus types 1 and 2 are neurotropic viruses that can be reactivated after a surgical or stressful intervention. Although such cases are uncommon, consequences can be debilitating, and variable treatment responses merit consideration. We describe a 41-year-old male with a history of varicella-mediated skin eruptions, who presented with continuing right arm pain, burning, and numbness in a C6 dermatomal distribution following a C5-6 anterior cervical discectomy and fusion and epidural steroid injections. The operative course was uncomplicated and he was discharged home on postoperative day 1. Approximately ten days after surgery, the patient presented to the emergency department complaining of severe pain in his right upper extremity and a vesicular rash from his elbow to his second digit. He was started on Acyclovir and discharged home. On outpatient follow-up, his rash had resolved though his pain continued. The patient was started on a neuromodulating agent for chronic pain. This case adds to the limited literature regarding this rare complication, brings attention to the symptoms for proper diagnosis and treatment, and emphasizes the importance of prompt antiviral therapy. We suggest adding a neuromodulating agent to prevent long-term sequelae and resolve acute symptoms.

## 1. Introduction

Varicella-zoster virus (VZV), herpes simplex virus type 1 (HSV-1), and herpes simplex virus type 2 (HSV-2), members of the Herpes virus family, are a group of neurotropic viruses that infect neurons, remain dormant in dorsal root ganglia, and by axoplasmic transport, can become reactivated during times of stress or immunocompromised states in the axonal terminals causing neuralgia, and a cutaneous rash most often in a segmental or radicular distribution. Although afferent sensory neurons are most often affected, motor neuron involvement has been reported in 0.5–31% cases of Herpes Zoster [1]. After a major surgical intervention, when the body is considered to be under significant stress or relative immunocompromised state, there have been a few reported cases of VZV-mediated radiculitis. Although these cases occur rarely, under various circumstances and with differing symptoms on presentation, the debilitating features and variable responses to treatment are a cause for concern and focused consideration (Table 1).

## 2. Case Presentation

We present the case of a 41-year-old male with a history of herpetic shingles, who had previously undergone a C5-6 foraminotomy two years prior to presentation, which had failed to relieve his right arm pain. Of note, after this surgery he experienced a recurrence of his shingles, which subsequently resolved. He presented to our clinic with continued pain and numbness in a C6 dermatomal distribution, as well as a burning sensation in this distribution, despite the previousely mentioned intervention. Conservative management including epidural steroid injections had failed to improve his symptoms.

On physical examination, he demonstrated full strength in all muscle groups bilaterally. He had no evidence of myelopathic signs. MRI of the cervical spine upon initial presentation to our clinic showed degenerative disk disease at C5-6, with bilateral foraminal stenosis.

Given that he had already undergone attempted foraminal decompression posteriorly, it was decided to proceed with C5-6 anterior cervical discectomy and fusion. The operative course was uncomplicated, the patient tolerated the procedure well, and he was discharged home on postoperative day 1.

**Figure 1:**
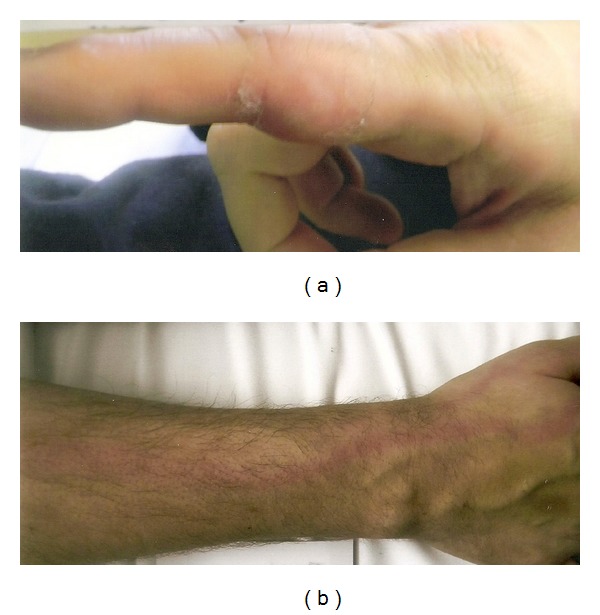
Postoperative photograph of the patient's index finger (a) and forearm (b) demonstrating a vesicular rash.

Approximately ten days after the procedure, the patient presented to the emergency department with complaints of severe pain in his right upper extremity and a vesicular rash from his elbow to his second digit (Figure 1). This was consistent with a herpetic shingles outbreak. He was subsequently started on Acyclovir and was discharged home.

At the follow-up visit, his vesicular rash had completely resolved. However, the upper extremity pain, which he had preoperatively, continued. Therefore, the possibility of neuropathic rather than radiculopathy was raised. He was started on neuromodulating agents including Lyrica and nortriptyline for chronic neuropathic pain.

## 3. Discussion

### 3.1. Time Period until Occurrence of Cutaneous Symptoms with Characteristic Skin Lesions

In the cases we reviewed, patients manifested VZV or HSV outbreaks from 2 days to 10 months after the inciting incident, which ranged from an epidural steroid injection to surgical manipulation. However, after surgical intervention, we noted that outbreaks or its resulting deficits occurred most often (9/13 cases) at 2–7 days postoperatively [2, 4, 5, 7, 9]. The correlation between onset of vesicular rash, symptoms, and time course postoperatively may be associated with the changing microenvironment and peak of the inflammatory response that can variably alter the latency of the viral particles in their respective dorsal root ganglion. Patients had resolution of symptoms attributable to Herpes ranging from days to 1 year, with complete to partial resolution of symptoms. 

### 3.2. Pathogenesis and Treatment of VZV of HSV Outbreaks

In many cases, surgical manipulation of nerve roots led to the development of shingles or herpes radiculitis in dermatomal distribution of the same manipulated nerve roots or those in adjacent levels [4, 5, 7, 9].

A recent case report demonstrated the successful usage of epidural corticosteroid injections in the vicinity of the affected spinal cord root in a patient with VZV-mediated radiculitis [1]. The proposed mechanism for symptom resolution is suppression of the intense inflammatory response surrounding the nerves. On the other hand, steroid injections of nerve roots involved in treating radicular pain may be implicated in causing VZV reactivation [3]. There have been several reported cases where reactivation is manifested after using selective nerve root or epidural injections of steroids [3, 6, 8]. Interestingly, a recent Cochrane review showed no demonstrated benefit of oral, intramuscular, or intravenous corticosteroids in preventing the development of postherpetic neuralgia [10].

We encountered several cases of herpetic outbreaks following craniospinal interventions that utilized different treatments with variable success (Table 2). Acyclovir and Valacyclovir have been used for both HZV and VZV resulting in resolution of lesions and prevention of postherpetic neuralgia [3, 5–8]. Cases with radiculitis have been treated with Vidarabine or IV Acyclovir, and while skin lesions and CSF pleocytosis resolved, neurologic status did not initially change [2, 4]. In another case, postoperative shingles was treated with IV Acyclovir, but following development of new lesions on different body sites at 48 hours, the patient was placed on Famciclovir and Gabapentin. Although skin lesions resolved, residual pain persisted at 1-year follow-up [5]. 

In a case series of postoperative neurosurgical patients who developed VZV or HSV reactivation, oral or IV Acyclovir was used successfully in the resolution of symptoms and lesions [9]. Interestingly, topical Acyclovir was also used to effectively treat two cases of VZV lesions [9]. Finally, in the one ophthalmic case encountered in this series and the entire literature review, viroptic ophthalmic solution was prescribed and completely resolved HSV dendritic keratitis in a postoperative neurosurgical patient. 

### 3.3. Neuromodulating Agents in the Treatment of Herpes Radiculitis

In the reviewed cases, although the herpetic outbreaks were treated with an appropriate antiviral agent either topically, orally, or intravenously, only a few tried to prevent postherpetic neuralgia with a neuromodulator, that is, amitriptyline and/or gabapentin [3, 5, 9]. Gabapentin has shown efficacy in the short-term, that is, less than two months, in preventing postherpetic neuralgia [11] and amitryptiline, which was started immediately after shingles outbreak in conjunction with an antiviral agent in elderly patients at a daily dose of 25 mg has been demonstrated to decrease pain prevalence in patients [12]. Therefore, it was surprising and noteworthy that these agents were not used more frequently in the cases we reviewed.

## 4. Conclusion

VZV- and HSV-mediated radiculitis and associated lesions are rare events which occasionally occur after surgical manipulation or steroid injections in the spinal canal. In cases with successful resolution, oral or intravenous antiviral agents, most commonly Acyclovir, have been used. Neuromodulating agents such as gabapentin and/or amitriptyline were used in some cases to prevent or decrease the severity of postherpetic neuralgia. 

This case report adds to the limited literature regarding this rare complication, brings attention to the symptoms for proper diagnosis and treatment, and emphasizes the importance of prompt antiviral therapy. We suggest the addition of a neuromodulating agent to prevent long-term sequelae and resolve acute symptoms.

## Figures and Tables

**Table 1:** Patients with herpes radiculitis—study, demographics, and presenting symptoms.

Author	Age/sex	Indication for initial intervention	Location	Presenting symptom	Time to outbreak, if applicable
Conliffe et al. [1]	75/M	L5 radiculopathy with foot drop	Lumbosacral distribution, L5	Herpetic rash	No information
Haverkos et al. [2]	52/M	T9 to L1 laminectomy for excision of arteriovenous malformation	Below umbilicus (T10) in all dermatomes.	Back pain and complete paralysis of both legs	9 days postoperatively
Haverkos et al. [2]	50/F	L4 laminectomy, L4-5 discectomy, and bilateral L5 foraminotomies for herniated disc	S2 and S3	Clusters of vesicular lesions in right S2 and S3 dermatomes	3 days postoperatively
Haverkos et al. [2]	55/F	L4-5 discectomy	Right buttock laterally	3 clusters of vesicles (3 × 4 cm)	5 days postoperatively
Makkar et al. [3]	30/F	Bilateral transforaminal fluoroscopy-guided steroid injection at L4 for 1 year of low back pain and radiculopathy	T10	Burning sensation and eruption of herpes zoster lesions	5 days after injection
Makkar et al. [3]	42/F	Serial transforaminal steroid injections for L5 radiculopathy of 6 months duration	T8	Pruritis and herpes zoster lesions	After third injection
Grauvogel and Vougioukas [4]	56/M	Foraminotomy for dysesthetic pain in C8 nerve root distribution	C6 and C7	New motor deficits	2 days postoperatively
Godfrey et al. [5]	59/M	2-stage anterior and posterior spinal fusion for progressive idiopathic adult lumbar scoliosis	T4 and T5	Severe left-sided chest pain and painful vesicular rash	29 days postoperatively
Godfrey et al. [5]	47/F	Anterior left 8th rib thoracotomy and cord decompression for lower extremity hyperreflexia and left T7 and T9 intercostal hyperalgesia	Left 7th, 8th, and 9th rib	Sudden onset severe left thoracogenic pain and vesicular eruptions	5 days postoperatively
Parsons and Hawboldt [6]	42/M	Series of 6 epidural blocks for complex regional pain syndrome	Right L2	Burning sensation followed by shingles lesions 7 days postoperatively	7 days postoperatively
Hung et al. [7]	70/M	Hospital admission for multiple fractures and compression of S1 ventral ramus	S1	Severe tingling pain and allodynia	30th day of hospital admission
Szokol and Gilbert [8]	52/F	Epidural steroid injection at L4-L5 interspace	S3	Pruritus in tailbone and herpes zoster outbreak in S3 distribution with no tenderness	4 days after injection
Nabors et al. [9]	57/F	T6-T8 laminectomy for spinal cord meningioma	Both buttocks	Vesicular rash	7 days postoperatively
Nabors et al. [9]	61/F	Occiput to C3 fusion because of underlying multiple myeloma	Vertex to base of neck with lesions at the rim of the pinna of the ear	Vesicular rash on the back of the head	5 months postoperatively
Nabors at el. [9]	63/F	Right L5 hemilaminectomy for excision of ruptured L5-S1 disc with topical corticosteroid applied to S1 nerve root	Left buttock	Vesicular rash	2 days postoperatively
Nabors et al. [9]	61/F	L3-L5 laminectomy for decompression with topical steroids applied to nerve roots	Both buttocks	Vesicular lesions	7 days postoperatively
Nabors et al. [9]	77/F	C3-C6 laminectomy for decompression of canal stenosis	Right side of lower back and buttocks	Vesicular rash	7 days postoperatively
Nabors et al. [9]	62/F	Right frontal craniotomy for clipping of aneurysm	Right side of chest	Vesicular rash	7 days postoperatively
Nabors et al. [9]	60/M	Left craniotomy for Glioblastoma Multiforme resection	Both eyes (V1 distribution)	Progressive right side weakness and, one week after, bilateral periorbital swelling	10 months postoperatively

**Table 2:** Treatments and outcome of patients with herpes radiculitis.

Author	Treatment	Follow-up and outcome, if applicable
Conliffe et al. [1]	Fluoroscopically guided R L5 transforaminal epidural injection with Depo-Medrol and lidocaine	6 weeks: pain decreased and improvement in right foot weakness with ability to ambulate
Haverkos et al. [2]	Vidarabine	Several days; skin lesions and CSF pleocytosis resolved but neurologic status unchanged
Haverkos et al. [2]	No treatment mentioned	Not mentioned
Haverkos et al. [2]	No treatment mentioned	Not mentioned
Makkar et al. [3]	Acyclovir	Lesion resolved and postherpetic neuralgia did not occur
Makkar et al. [3]	Acyclovir and amytryptyline	Few days; resolution of skin lesions postherpetic neuralgia did not occur
Grauvogel and Vougioukas [4]	Acyclovir	3 months: neurologic deficits improved
Godfrey et al. [5]	Acyclovir (then switched to famciclovir) and gabapentin	1 year: pain resolution with mild rib pain (3/10) and no new skin lesions
Godfrey et al. [5]	Valacyclovir and gabapentin (GBN later discontinued due to adverse effects)	1 year: no pain or skin eruptions
Parsons and Hawboldt [6]	Valacyclovir	1 year: resolution of complex region pain syndrome after 12-month period
Hung et al. [7]	Valacyclovir	7 days: skin lesions and tingling pain resolved
Szokol and Gilbert [8]	Acyclovir	Several weeks; skin lesions resolved and patient had 50% reduction of radicular symptoms
Nabors et al. [9]	Acyclovir	Complete resolution of rash and skin lesions
Nabors et al. [9]	Antibiotics and Acylovir; tricyclic antidepressant and analgesics administered for postherpectic neuralgia	9 days: resolution of skin lesions but patient developed tenderness at site of former lesions with relief provided by TCA and analgesics
Nabors at el. [9]	Acyclovir	Resolution of skin lesions
Nabors et al. [9]	Acyclovir	Resolution of skin lesions
Nabors et al. [9]	Acyclovir	Resolution of skin lesions
Nabors et al. [9]	No treatment mentioned	Patient transferred to another hospital
Nabors et al. [9]	Virotropic ophthalmic solution	Resolution of Herpes dendritic keratitis but patient became comatose and eventually died
